# The Effect of Recruitment Maneuver on Static Lung Compliance in Patients Undergoing General Anesthesia for Laparoscopic Cholecystectomy: A Single-Centre Prospective Clinical Intervention Study

**DOI:** 10.3390/medicina60040666

**Published:** 2024-04-19

**Authors:** Nada Anđelić, Arsen Uvelin, Edita Stokić, Radmila Popović, Ranko Zdravković, Andrej Preveden, Nenad Zornić

**Affiliations:** 1 Clinic for Anesthesia, Intensive Care and Pain Medicine, Clinical Centre of Vojvodina, 21000 Novi Sad, Serbia; andjelicnada2@gmail.com (N.A.); radmila.popovic@mf.uns.ac.rs (R.P.); 2 Faculty of Medical Sciences, Kragujevac, University of Kragujevac, 34000 Kragujevac, Serbia; nenadzornic@gmail.com; 3 Faculty of Medicine, Novi Sad, University of Novi Sad, 21000 Novi Sad, Serbia; edita.stokic@mf.uns.ac.rs (E.S.); ranko.zdravkovic@mf.uns.ac.rs (R.Z.); andrej.preveden@mf.uns.ac.rs (A.P.); 4 Clinic for Endocrinology, Diabetes and Metabolism, Clinical Centre of Vojvodina, 21000 Novi Sad, Serbia; 5 Institute of Cardiovascular Diseases of Vojvodina, 21204 Sremska Kamenica, Serbia; 6 Department of Surgery, Clinical Centre of Kragujevac, 34000 Kragujevac, Serbia

**Keywords:** laparoscopic cholecystectomy, alveolar recruitment maneuver, static lung compliance

## Abstract

*Background and Objectives*: The aim of this study was to examine whether the use of an alveolar recruitment maneuver (RM) leads to a significant increase in static lung compliance (Cstat) and an improvement in gas exchange in patients undergoing laparoscopic cholecystectomy. *Material and Methods*: A clinical prospective intervention study was conducted. Patients were divided into two groups according to their body mass index (BMI): normal-weight (group I) and pre-obese and obese grade I (group II). Lung mechanics were monitored (Cstat, dynamic compliance—Cdin, peak pressure—Ppeak, plateau pressure—Pplat, driving pressure—DP) alongside gas exchange, and hemodynamic changes (heart rate—HR, mean arterial pressure—MAP) at six time points: T1 (induction of anesthesia), T2 (formation of pneumoperitoneum), T3 (RM with a PEEP of 5 cm H_2_O), T4 (RM with a PEEP of 7 cm H_2_O), T5 (desufflation), and T6 (RM at the end). The RM was performed by increasing the peak pressure by +5 cm of H_2_O at an equal inspiration-to-expiration ratio (I/E = 1:1) and applying a PEEP of 5 and 7 cm of H_2_O. *Results*: Out of 96 patients, 33 belonged to group I and 63 to group II. An increase in Cstat values occurred after all three RMs. At each time point, the Cstat value was measured higher in group I than in group II. A higher increase in Cstat was observed in group II after the second and third RM. Cstat values were higher at the end of the surgical procedure compared to values after the induction of anesthesia. The RM led to a significant increase in PaO_2_ in both groups without changes in HR or MAP. *Conclusions*: During laparoscopic cholecystectomy, the application of RM leads to a significant increase in Cstat and an improvement in gas exchange. The prevention of atelectasis during anesthesia should be initiated immediately after the induction of anesthesia, using protective mechanical ventilation and RM.

## 1. Introduction

In recent years, the question of whether the protective strategy of lung ventilation and the application of the RM should become a strategy of mechanical ventilation (MV) during general anesthesia has been discussed [1]. The term “protective”, in the context of MV, means a reduction in factors that can lead to ventilator-induced lung injury. The intraoperative strategy of protective ventilation involves the use of low tidal volumes (Vt ≤ 8 mL/kg of ideal body weight) in combination with positive-end expiratory pressure (PEEP) and the occasional use of the RM [2].

During general anesthesia, limited atelectasis occurs in almost all patients (90%) [3,4]. This is the result of a decrease in chest muscle tone and a reduction in functional residual capacity (FRC) [5]. This effect of anesthesia is more pronounced in patients with increased body mass due to the weight of the chest wall and the compression of the lungs by the abdominal organs. In laparoscopic abdominal procedures, the effects of pressure on the lungs and reduction in lung compliance and FRC are a consequence of pneumoperitoneum formation [6,7,8].

In clinical practice, respiratory mechanics is a key element for the monitoring of mechanically ventilated patients and guiding mechanical lung ventilation [2]. Changes in respiratory mechanics, such as lower static lung compliance (Cstat) values and low oxygenation indices, are both good prognostic indicators of the risk of death [9]. High driving pressure (DP) is also associated with an increased risk of death in patients with acute respiratory distress syndrome (ARDS) [10,11]. The monitoring of respiratory mechanics is an everyday practice in ICU patients. These data reinforce the importance of measuring lung mechanics indices during general anesthesia. 

The objectives of this study are as follows:

The primary goal of our research was to determine the impact of this newly developed RM on Cstat in patients who undergoing laparoscopic cholecystectomy under general anesthesia.

The secondary objectives were to determine whether there would be differences in the change in Cstat between obese and normal-weight individuals if the RM would cause hemodynamic changes in HR and MAP in both groups of subjects, as well as the probable influence of the RM on changes in partial pressure of oxygen in arterial blood (PaO_2_).

## 2. Materials and Methods

The study was conducted as a clinical prospective open-label intervention study, approved by the Ethics Committee of the Clinical Centre of Vojvodina, Novi Sad, Serbia, number 00-37, dated 9 February 2023. The study protocol complied with the Declaration of Helsinki, and the procedures were performed after obtaining the written informed consent of all participants. The study is registered with ClinicalTrials.gov, NCT06256029, accessed on 13 February 2024.

### 2.1. Patient Selection

The study was performed in patients over 18 years of age, with an American Society of Anesthesiologists Physical Status ≤ 3 (ASA), who were scheduled for laparoscopic cholecystectomy, with the minimal duration of the procedure being 45 min.

The patients were divided into two groups. Group I consisted of normal-weight patients (body mass index—BMI from 18.5 to 24.9 kg/m^2^), while group II consisted of patients with increased body mass (pre-obese and obese class I with BMI from 25 to 34.9 kg/m^2^). Randomization was not performed since the patients were divided into two groups according to BMI, and every patient who met the inclusion criteria was part of the study. 

In total, 96 patients participated in the study; 33 participants were normal weight (group I), and 63 were pre-obese and obese class I (group II). This study did not include patients under the age of 18, pregnant women, patients with an ASA status > 3, BMI < 18.5, and ≥35 kg/m^2^, patients who previously had open abdominal surgery, previous lung surgery, obstructive or restrictive lung diseases, neuromuscular diseases and procedures lasting less than 45 min.

Patients who developed hemodynamic instability during the use of the RM (a drop in systolic blood pressure—SBP—by more than 20% compared to basal values or SBP < 90 mmHg), bradycardia (a drop in HR by more than 20% or HR < 50/min.), or a decrease in saturation (SpO_2_ ≤ 92% for more than 1 min) were recorded and excluded from the final analysis. Patients who withdrew their informed consent were also excluded from the study, as well as in the case of conversion to open surgery.

Patient recruitment and anthropometric measurements were all performed during their preoperative assessment.

### 2.2. Anesthesia Technique

Midazolam (0.02 mg/kg i.v.) and fentanyl (1 mcg/kg i.v.) were used for the coinduction of general anesthesia. After preoxygenation (100% oxygen, 6 L/min, 5 min, via facemask), propofol at 2 mg/kg was administered for induction. Anesthesia was maintained with sevoflurane, with a targeted minimal alveolar concentration (MAC) of 0.8–1.0. Analgesia was provided with fentanyl (3–5 mcg/kg), neuromuscular relaxation with rocuronium (0.6 mg/kg for intubation and a maintenance dose of 0.1 mg/kg), and the reversal of neuromuscular blockade with neostigmine.

During anesthesia, standard monitoring was used, which included non-invasive blood pressure (NIBP), electrocardiography (EKG), pulse oximetry (SpO_2_), capnography (etCO_2_), and nasopharyngeal temperature (°C), (Infinity XL monitor, Dräger, Lübeck, Germany). The Bispectral index monitoring (BIS, Medtronic, Covidien, Minneapolis, MN, USA) was used to assess the depth of anesthesia and was maintained between 40 and 60, and the training of four responses (TOF) was used to assess muscle relaxation. Intra-abdominal pressure during the laparoscopic procedure and all of the RMs was 12 ± 1 mmHg.

Anesthesia was delivered with the Dräger Primus anesthesia machine, using volume-controlled mechanical ventilation (VCV) with a fresh gas flow of 2 L/min, FiO_2_ at 0.5, and Vt at 7 mL/kg of ideal body weight (IBW—male: 50 + {0.91 × [height in cm − 152.4]} female: 45.5 + {0.91 × [height in cm − 152.4]}) [12], with an initial PEEP of 3 cm H_2_O. The inspiratory-to-expiratory ratio was 1:2 (I/E-1:2), with an initial respiratory rate of 12 breaths per minute. The targeted end-tidal CO_2_ (etCO_2_) was 32–37 cm H_2_O, which was adjusted by changing the respiratory rate. Before every RM, a peak pressure (Ppeak) was measured during VCV and noted. For the purpose of the RM, inspiratory pressure for the RM was calculated by adding 5 cm of H_2_O to the Ppeak that was measured in the previous step.

### 2.3. Recruitment Maneuver

Recruitment maneuvers were performed by changing the ventilation mode to pressure-controlled ventilation (PCV); inspiration pressure for the recruitment was calculated as described above, changing the I/E ratio from 1:2 to 1:1 and setting the positive end-expiratory pressure (PEEP) values of 5 cm of H_2_O during the first RM and 7 cm of H_2_O during the second and third RMs. The respiratory rate during the RM was set at 10 breaths per minute, with the RM lasting 30 s (5 breaths, each inspiration lasting 3 s, followed by 3 s of expiration). In both patient groups, RM was performed three times: the first time after the formation of pneumoperitoneum and positioning the patient in the reverse Trendelenburg position (PEEP of 5 cm H_2_O), the second time during the surgery (PEEP of 7 cm H_2_O) and the third time at the end of the surgery, just before waking the patient (PEEP of 7 cm H_2_O). After each increase in PEEP and RM, the ventilation was then switched back to VCV with the initial values of Vt, I/E, and targeted etCO_2_, except for the higher PEEP that was set during the RM. The RM used in this study was designed by us. It was developed three months before the start of our research and performed on patients undergoing laparoscopic cholecystectomy. 

### 2.4. Hemodynamic and Respiratory Monitoring

HR, MAP, and SpO_2_ were monitored during all stages of the laparoscopic cholecystectomy, as well as after the RM, and the parameters of respiratory mechanics were measured in the next time points as follows: (T1) after the induction of anesthesia and before the formation of pneumoperitoneum; (T2) after the formation of pneumoperitoneum and the positioning of the patient in the reverse Trendelenburg position; (T3) after RM with PEEP values of 5 cm H_2_O; (T4) after RM with PEEP values of 7 cm H_2_O; (T5) after desufflation; (T6) at the end of the operation, just before waking, after the RM and PEEP values of 7 cm H_2_O. All measurements were made in the VCV mode of mechanical ventilation, and RM was performed in the pressure control ventilation (PCV) mode of mechanical ventilation.

The parameters of the lung respiratory mechanics that were measured were the Ppeak, plateau pressure (Pplat), dynamic compliance (Cdin), Cstat, and DP. The measurements were taken in dynamic conditions (Ppeak and Cdin) and using the maneuver of extending the inspiratory pause to 40% of the length of inspiration; in static conditions, Pplat and Cstat values were obtained [12]. All measurements were directly visible on and taken from the Dräger Primus anesthesia machine screen during mechanical ventilation. DP was calculated according to the formula DP = Pplat − PEEP [13].

The measurements of MAP and HR in the mentioned six time points (T1–T6), especially the values immediately after the RM, were the ones that would be taken into analysis. Arterial blood gas analysis was sampled two times during the surgical procedure, before the first RM, and after the second RM. 

### 2.5. Statistical Analysis

The arithmetic means with standard deviation or the medians, including minimum and maximum values, were used to describe continuous numerical attributes as measures of central tendency. Attribute characteristics were described using frequency distributions and percentages, as well as absolute and relative numbers. 

To test the statistical significance of categorical variables, the χ^2^ test of independence or Fisher’s exact test was used. In addition, Yates correction was applied for the χ^2^ test of independence for 2 × 2 tables. For comparing continuous variables of two different groups, Student’s *t*-test for independent samples or its non-parametric alternative, the Mann–Whitney U test, was used. If repeated measurements were performed within the same group, the paired sample Student’s *t*-test or its non-parametric alternative, the Wilcoxon signed-rank test, was used.

For determining the significance of changes in a parameter measured at multiple time points, repeated measures analysis of variance (ANOVA) or its non-parametric alternative, the Friedman test, was used. Bonferroni correction was applied for the post hoc testing of significant changes in ANOVA, while the Wilcoxon signed-rank test was employed for Friedman analysis. 

The statistical analysis was performed using SPSS version 26 (IBM SPSS, Armonk, NY, USA), MedCalc version 20.115 (MedCalc Software Ltd., Ostend, Belgium), and Microsoft Excel 2019 (Microsoft, Redmond, WA, USA). The results were presented in tables and graphs, and statistical significance was considered at an alpha level of *p* < 0.05. 

## 3. Results

### 3.1. Anthropometric and Demographic Characteristics of the Participants

A total of 96 patients were included in our study, with 33 patients belonging to the group of normal-weight individuals (group I) and 63 patients belonging to the group of overweight and obese individuals (group II). The majority of patients in group I—60.6%—and group II—66.7%—were women (Table 1). 

### 3.2. Parameters of Respiratory Mechanics

The parameters of respiratory mechanics were monitored at six time points during the laparoscopic cholecystectomy and are shown in Table 2. 

T1—By monitoring the parameters of respiratory mechanics after the induction of anesthesia, statistically significant differences were identified between the subjects of group I and group II. Subjects in group I had statistically significant lower values of Ppeak, Pplat, and DP and statistically significant higher values of Cdin and Cstat (*p* ≤ 0.001).

T2—After creating the pneumoperitoneum and positioning the patient, there was an increase in the values of Ppeak, Pplat, and DP and a decrease in the values of Cdin and Cstat in both groups of subjects with statistically significant differences between the groups regarding the studied variables (*p* ≤ 0.001). Ppeak, Pplat, and DP were significantly lower, and the values of Cdin and Cstat were statistically significantly higher in group I subjects.

T3 and T4—After performing the RM with PEEP values of 5 cm H_2_O and 7 cm H_2_O, a statistically significant difference was found in the obtained results regarding Ppeak, Pplat, Cdin, Cstat, and DP between the investigated groups. After both recruitments, there was an increase in the values of Ppeak, Pplat, and DP, with statistically higher values in the overweight and obese groups compared to the normal-weight subjects. It is noteworthy that there was also an increase in the values of Cdin and Cstat in both groups of subjects, with statistically significant lower values in the overweight and obese group compared to the normal-weight subjects.

T5—After the desufflation of the pneumoperitoneum, there was a decrease in the values of Ppeak and Pplat compared to the previous measurement point, but an increase in the values of Cdin and Cstat in both groups with statistically significant differences in the obtained results between the investigated groups (*p* ≤ 0.001).

T6—After the third RM with a PEEP of 7 cm H_2_O, at the end of the operation, there was an increase in lung compliance (Cdin and Cstat) compared to the values after desufflation in both groups of subjects, with a decrease in Ppeak, Pplat, and DP and values of Cdin and Cstat being significantly higher in the normal-weight group, and Ppeak, Pplat, and DP appearing significantly lower compared to the overweight and obese group (group II). 

**Figure 1:**
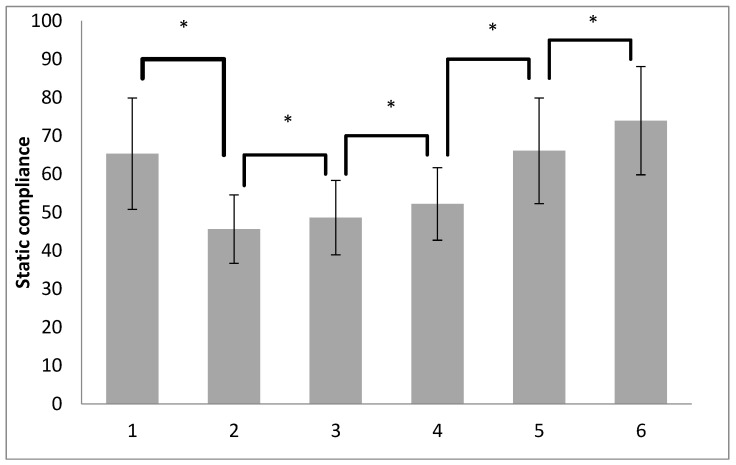
Post-hoc analysis of static compliance of both groups measured at 6 time points.

Statistically significant changes in the value of Cstat in subjects were determined at the investigated time points by repeated measures of analysis of variance (ANOVA) for both groups (Figure 1).

Additional (post hoc) analysis revealed between which time points a statistically significant change in Cstat values occurred for both groups of subjects combined (decrease or increase). It is observed (Figure 1) that Cstat is significantly higher before pneumoperitoneum compared to the value after pneumoperitoneum. Furthermore, Cstat is higher after the RM with a PEEP of 5 cm H_2_O compared to the value after pneumoperitoneum. Until the end of the surgical procedure, there is a continuous increase in Cstat at all time points up to the last time point (RM with a PEEP of 7 cm H_2_O at the end of the operation). The changes in Cstat values at all time points were statistically significant (*p* < 0.001).

**Figure 2:**
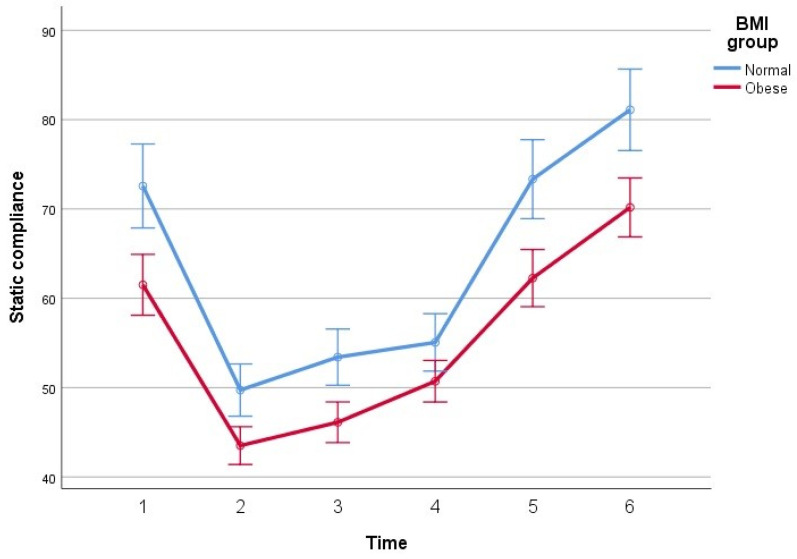
Static lung compliance of group I and group II measured at 6 time points ($\bar{x}$ ± 95% CI).

When comparing group I and group II subjects at each time point, the Cstat value is higher in normal-weight subjects than in obese subjects. The largest difference between the groups is observed after deflation (point 5, Figure 2) and then after RM with a PEEP of 7 cm H_2_O at the end of the operation (point 6, Figure 2).

The greatest change in Cstat occurred after creating the pneumoperitoneum (∆2–∆1) and then after deflation (∆5–∆4) in both groups of patients (Table 3).

During the time Δ4–∆3 (after the second RM with a PEEP of 7 cm H_2_O) and Δ6–∆5 (after the third RM at the end of the operation), the change in Cstat was higher in the group of overweight and obese patients compared to the normal-weight group. Cstat values were higher at the end of the operation after the last RM compared to the values after the induction of anesthesia in both groups of subjects.

### 3.3. Gas Exchange

Regarding the values of PaO_2_, the P/F ratio, PaO_2_/PAO_2_, and the alveolar-arterial oxygen gradient (A-aDO_2_), a statistically significant difference was observed between group I and group II subjects at both monitoring times before and after RM which is shown in the Table 4. There was no statistically significant difference in PaCO_2_ values between the groups (t = 0.756; *p* = 0.452).

Using Student’s *t*-test, the change in the partial pressure of oxygen was estimated for subjects from both groups together. A statistically significant increase in the partial pressure of oxygen was found in subjects from both groups from the moment before moment before performing the RM (168.76 ± 48.73) to the moment after performing the RM (186.81 ± 41.62), t (95) = 8.964, *p* < 0.001 (Table 5).

The average increase in the PaO_2_ value was 18.05, with a 95% confidence interval ranging from 14.05 to 22.05. The value of eta squared (0.46) indicated that the impact of the intervention was very large.

### 3.4. Hemodynamic Parameters

A one-way analysis of variance of repeated measures was used to compare HR values at six measurement points. Table 6 presents the measures of central tendency, including the mean, standard deviation, and 25th, 50th, and 75th percentiles. The test revealed a statistically significant change in HR values in the investigated time intervals, Wilks’ lambda = 0.369, F (5.91) = 31.177, *p* < 0.001. The multivariate eta squared was 0.631, which, according to Cohen’s criteria, represents the very large effect of the intervention.

**Figure 3:**
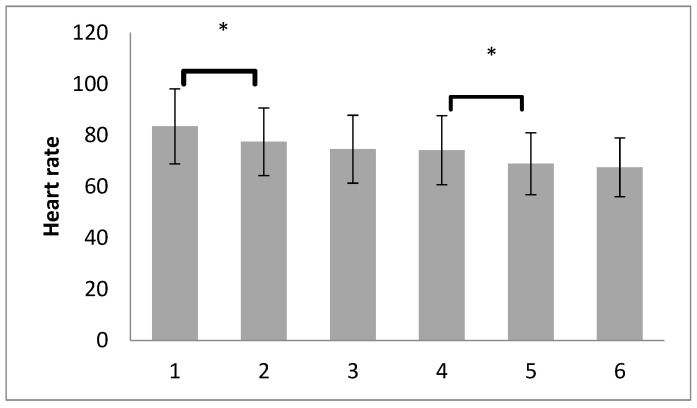
Post hoc (Bonferoni) analysis of HR in both groups at different time points (mean ± SD), * *p* < 0.001.

In Figure 3, it can be observed that the HR value is significantly lower after the pneumoperitoneum formation compared to before the pneumoperitoneum (*p* < 0.001; 95% CI = 2.606–9.435). A statistically significant decrease in HR was also observed after deflation compared to the value after RM with a PEEP of 7 cm H_2_O (*p* < 0.001; CI = 2.576–7.924). It should be noted that none of the RMs led to a statistically significant change in HR compared to the previous time point. All observed changes in HR are the result of pneumoperitoneum formation or deflation.

Since not all conditions for performing a one-way analysis of variance of repeated measures were met, a non-parametric alternative, the Friedman test, was used to compare the values of MAP among subjects from both groups, (Table 7).

The values of MAP measured at six specified time points were compared. The Friedman test revealed a statistically significant change in MAP values in the investigated time intervals, χ^2^ (5, *n* = 96) = 63.241, *p* < 0.001. An examination of the medians (50th percentile) showed an increase in MAP values up to the point after the RM with a PEEP of 5 cm H_2_O, followed by a decrease until the last measurement point, after the RM, with a PEEP of 7 cm H_2_O at the end of the operation.

**Figure 4:**
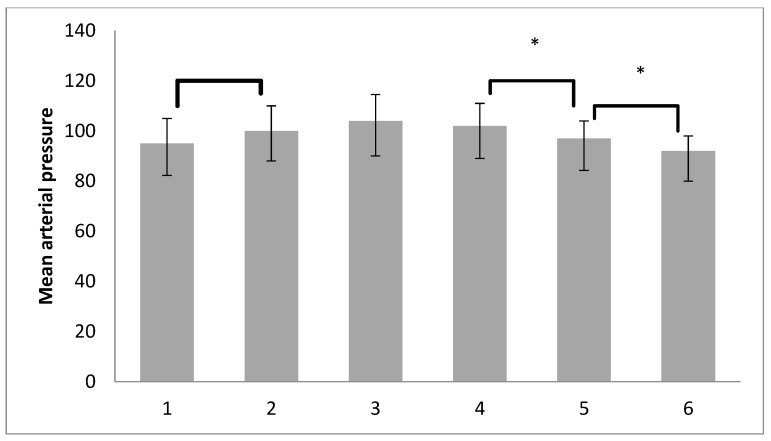
Post hoc (Bonferoni) analysis of MAP of both groups at different time points (median; 25–75. percentile), * *p* < 0.01.

To determine which time points had statistically significant differences in MAP values, post hoc analysis was conducted using the Wilcoxon rank-sum test with Bonferroni correction. Five pairwise Wilcoxon tests were used to compare the time points, so the revised alpha level of statistical significance was set at 0.01. On Figure 4 it can be observed that the MAP value is significantly higher after the pneumoperitoneum formation compared to before pneumoperitoneum (*p* = 0.008; z = −2.646) with a small effect size according to Cohen’s criteria (r = 0.19). Next, a statistically significant decrease in MAP values was observed after the desufflation compared to the RM with a PEEP of 7 cm H_2_O (*p* = 0.001; z = −3.210) with a small effect size (r = 0.23). Finally, a decrease in MAP values was noted after RM at the end of the operation compared to the value after desufflation (*p* < 0.001; z = −3.783) with a small effect size (r = 0.27).

## 4. Discussion

### 4.1. Respiratory Mechanics

During anesthesia, volumetric computed tomography (CT) analysis of the lungs revealed that in the supine position, atelectasis develops in almost all patients, mainly in the dependent lower parts of the lungs [14]. Even in healthy individuals, atelectasis occurring during anesthesia leads to an increase in ventilation/perfusion mismatch and gas exchange abnormalities [15]. In obese individuals, lung volume is reduced due to the accumulation of visceral fat and increased abdominal volume [16]. Obesity is associated with reduced FRC, decreased lung compliance, and increased airway resistance [17]. With this said, the effects of anesthesia in obese individuals become even more pronounced [16]. Following anesthesia induction, the parameters of respiratory mechanics differed significantly between normal-weight and obese individuals in our study. Ppeak, Pplat, and DP values were significantly higher, while Cdin and Cstat values were significantly lower in the group with an increased BMI, consistent with changes in lung function in obese individuals during anesthesia. Pneumoperitoneum disrupts respiratory mechanics in normal-weight and obese individuals [18,19,20,21]. Such an effect of pneumoperitoneum is associated with a reduction in lung volume and the occurrence or worsening of existing atelectasis, as confirmed by CT analysis [22]. 

In the study by Sharma et al., it was observed that after pneumoperitoneum formation, Ppeak and Pplat can increase by more than 50% while simultaneously decreasing respiratory system compliance by 47% [23]. In the same study, after desufflation, Ppeak and Pplat remained elevated by 37% and 27%, respectively, compared to the values before pneumoperitoneum formation, while compliance remained at 87% of the pre-pneumoperitoneum value [23]. In this study, the RM was not applied to the study subjects. Tomescu et al. found that after anesthesia induction and pneumoperitoneum formation, obesity was the main risk factor for decreased lung compliance and increased Pplat. After pneumoperitoneum desufflation and the return of the patient to the supine position, the parameters of respiratory mechanics did not return to the baseline values [8]. The RM was also not applied in this study of Tomescu. The use of the RM is widely accepted in patients with ARDS in intensive care units, but the frequency of its use during anesthesia remains a question. While protective lung ventilation strategies are widely accepted during anesthesia, the utilization of the RM and individualization of PEEP are not as prevalent [1]. In our study, the RM protocol was initially designed to incorporate lower Ppeak values during inspiratory breaths, which was maintained throughout each RM, along with short inspiratory times. Both of these variables aim to preserve hemodynamic stability while simultaneously improving oxygenation and Cstat.

The effectiveness of the alveolar RM was monitored in our study based on changes in Cstat values, as well as changes in PaO_2_, before and after the RM. Of note, Cstat can also be used as a measure of the degree of lung damage [24]. The RM during anesthesia is not standardized as a procedure. Most studies compared patients who underwent the RM with those who did not (control group) [25]. However, from such available data, it was not possible to identify which RM was more effective. 

According to our study protocol, the RM consisted of increasing Ppeak by 5 cm H_2_O, changing the I/E ratio to 1:1 with the application of PEEP at 5 and 7 cm of H_2_O. In our study, the greatest change (reduction) in static lung compliance, from T1 to T6, occurred after pneumoperitoneum formation. Pneumoperitoneum led to a decrease in Cstat in both groups of subjects, with the note that Cstat was significantly lower in the obese group. The greatest increase in Cstat occurred after desufflation in both groups. At that point, Cstat values reached Cstat values after anesthesia induction, most likely due to the application of the RM.

The application of the RM was found to have a favorable effect on Cstat. All three RMs led to an increase in Cstat in both groups of subjects, with the third RM performed just before waking the patient having the greatest effect on increasing the Cstat values. This significant rise in the Cstat value at the end of the procedure is a result not only due to the desufflation of the pneumoperitoneum but also as a result of the repeated use of the RM. According to Almarakbi et al., repeated recruitment maneuvers, in combination with PEEP, have a favorable effect on Cdin and PaO_2_, leading to a decrease in PaCO_2_ [26]. The increase in Cstat after the second and third RM was higher in the pre-obese and obese group compared to the normal-weight subjects, indicating that their lungs were more recruitable. Lung recruitability is the ability of the lungs to open collapsed alveoli and improve gas exchange. The increase in Cdin and Cstat immediately after the RM indicates a reduction in atelectasis volume, which should then lead to an increase in PaO_2_ as a result of improved ventilation–perfusion (V/P) matching [8,9,10,11,12,13,14,15,16,17,18,19,20,21,22,23,24,25,26,27]. Respiratory system compliance is correlated with the volume of aerated lungs [28]. Lung recruitability in obese patients is similar to that of normal-weight patients (in COVID-19 patients with ARDS) [29]. In a study by Futier et al., during laparoscopic surgery with the use of low tidal volumes and different PEEP levels, the RM led to changes in end-expiratory lung volume (EELV) of 10% in normal-weight patients and 20% in obese patients, with a statistically significant difference between the groups [24]. Increasing EELV, in return, increases lung compliance, which is consistent with our results. Cstat, at the end of the operation, in both subject groups, had higher values compared to the values after anesthesia induction, which is again the result of repeated RM. The RM and PEEP, in the context of protective mechanical ventilation, should be used to protect the lungs, not just as a method to improve oxygenation [27]. Relatively low PEEP values of 5 and 7 cm of H_2_O used in our study, along with the RM, led to an increase in Cstat and improved gas exchange. According to Gattinoni et al., the PEEP value should be determined as a compromise between oxygenation, cyclic opening and closing of alveoli, and patient hemodynamic stability [30]. The PEEP values used in our study were likely not optimal for each individual participant, and even higher PEEP values should have been used for a more pronounced improvement in oxygenation and Cstat increase in some patients. The study of Mazzinari and colleagues focused on targeting PEEP based on intraabdominal pressure during laparoscopy [31]. Future studies should aim to determine the optimal combination of PEEP and intraabdominal pressure in terms of clinical outcomes. The individualization of PEEP should consider the individual characteristics of the patient, the type of surgical procedure, and the positioning of the patient during the surgery [32]. The goal of the individualization is to achieve the best oxygenation as well as the best parameters of the respiratory mechanics, such as minimal individual DP and maximizing the Cstat values [32,33]. 

### 4.2. Blood Gas Analyses

Blood gas analyses were performed immediately before and after the RM. Following the RM, a significant increase in PaO_2_ was found in both groups together (*p* < 0.001). The average increase in PaO_2_ after the RM was 18.05 mmHg, confirming the effectiveness of the performed RM. The level of PaO_2_ depends on the partial pressure of oxygen in the alveoli (P_A_O_2_), gas exchange mechanisms, the V/P ratio, the presence of atelectasis, intrapulmonary shunt, and hypoventilation [34]. PaO_2_ decreases during general anesthesia in obese patients compared to normal-weight individuals, and gas exchange abnormalities are directly related to BMI [20,35]. In our study, a correlation between PaO_2_ and BMI was also shown.

There was no statistically significant difference in PaCO_2_ between the two groups of subjects before and after the RM. PaCO_2_ levels are not correlated with BMI [17,20].

### 4.3. Hemodynamic Changes

Only a few studies have examined the impact of the RM on hemodynamic changes in patients during anesthesia. The effects of the RM depend on the way it is performed, the duration of the RM, the pressures applied during the RM, and the individual characteristics of the patients [36]. Changes in hemodynamics during the RM are a result of increased intrathoracic pressure, decreased venous return to the heart, decreased left ventricular volume at the end of the diastole, and decreased stroke volume (SV) [37]. The decrease in SV during the RM ranges from 20% to 43% [36,37,38]. The preoperative optimization of intravascular volume can reduce the unwanted hemodynamic effects of RM and PEEP application [7]. In our study, changes in HR measured at six time points revealed a statistically significant decrease in HR during pneumoperitoneum formation and after desufflation (*p* < 0.001). The changes in HR were not related to the RM. 

Monitoring changes in MAP at six time points also revealed a statistically significant decrease in MAP (*p* < 0.001) after pneumoperitoneum formation and after desufflation. After the third RM, there was also a significant decrease in MAP (*p* < 0.001), which may be related to the application of RM, with the small impact of the intervention on MAP. Since a third RM was applied at the end of the surgery, we cannot exclude that the cause of the decrease in MAP was due to a decrease in surgical stimulation at the end of the surgery. In any case, the change in MAP was not greater than 20% at any measurement point compared to values after anesthesia induction.

During the RM, there was no observed decrease in etCO_2_ values, and there were no changes in HR values. Indirectly, we can conclude that changes in MAP values were not clinically significant. MAP values remained within an acceptable range.

During the study, invasive blood pressure (IBP) monitoring was not used, so the precise data on the MAP levels at each moment of RM application were not recorded, which presents a limitation of our study.

### 4.4. Strengths and Limitations of the Study

The monitoring of Pplat and Cstat is not in routine use in operating rooms worldwide, nor is the performance of the RM. Our study adds to the current knowledge by demonstrating the safe use of the RM during general anesthesia facilitated by the design of the RM itself. Monitoring respiratory mechanics during general anesthesia is readily available with the use of modern anesthesia machines. We strongly believe that the RM could be performed routinely and safely and that the monitoring of Pplat and Cstat should become a standard practice in operating rooms. A major limitation of our study is the absence of a control group, which would have been the “standard of care, no recruitment maneuver” group. The groups in our study differed only by body weight, as we aimed to study differences in the effect of the RM between these patients, assuming the RM would have a positive impact on respiratory mechanics. Apart from that, the anesthesiologist performing the RMs was not blinded to the group, which could introduce bias. 

Our study found that the RM led to increased Cstat values and improved oxygenation. We believe that the benefits of the RMs, with lower Ppeak and shorter inspiratory times, were demonstrated through the monitoring of Cstat, which showed enhanced Cstat at each time point after the RM. The PEEP in our study was not individualized, although previous research suggests that obese patients undergoing laparoscopic surgery were found to have better oxygenation, lower DP, and redistribution of ventilation toward dependent lung areas measured by electrical impedance tomography and using individualized PEEP [39]. The impact of the RM on patient postoperative outcomes remains unclear. An analysis of the pooled data suggests that the RMs reduce postoperative complications [25]. The real benefit would be if the “RM group patients” are shown to have fewer postoperative respiratory complications and shorter hospital stays compared to the “standard of care patients without the RM”. Future research should explore the implementation of the RM in everyday practice during laparoscopic procedures, as well as optimal methods for performing the RM and determining the best PEEP settings.

## 5. Conclusions

The ventilation strategy for patients during anesthesia is of the utmost importance, especially in laparoscopic interventions for high-risk patient groups. Therefore, the prevention of atelectasis formation during anesthesia should begin immediately after induction, with the application of protective ventilation measures, small tidal volumes, moderate PEEP levels, and the occasional use of the RM. We have demonstrated that such an approach to mechanical lung ventilation during laparoscopic cholecystectomy leads to increased Cstat values, improved gas exchange, and minimal impact on MAP in both normally nourished and obese patients, making it a safe practice.

## Figures and Tables

**Table 1:** Anthropometric and demographic characteristics of the participants.

Variable (Factor)	Normal-Weight (*n* = 33)	Pre-Obese and Obese Class I (*n* = 63)	*p* Value
Gender *n* (%)			0.555
Male	13 (39.4)	21 (33.3)	
Female	20 (60.6)	42 (66.7)	
Age	46.91 ± 16.74	54.78 ± 13.21	0.013
Height (cm)	171.58 ± 9.01	168.00 ± 10.23	0.094
Weight (kg)	68.97 ± 9.45	83.70 ± 11.73	<0.001
BMI (kg/m^2^)	23.33 ± 1.51	29.58 ± 2.53	<0.001
Neck circumference (cm)	38.20 ± 9.59	39.79 ± 3.48	0.241
Waist circumference (cm)	82.52 ± 8.07	96.48 ± 10.35	<0.001
Hip circumference (cm)	95.45 ± 5.89	106.78 ± 9.67	<0.001
Waist/hip circumference ratio	0.86 ± 0.07	0.91 ± 0.07	0.011
ASA class *n* (%)			0.006
ASA—1	11 (33,3)	6 (9.5)	
ASA—2	19 (57.6)	40 (63.5)	
ASA—3	3 (9.1)	17 (27.0)	

Regarding the height of the subjects, there was no statistically significant difference between the groups, while the difference in body weight was set as a criterion for inclusion in this study. Subjects belonging to group II had statistically significant higher body mass and BMI (*p* < 0.001), as well as a statistically significant larger waist circumference than normal-weight subjects (t = 6.747; *p* < 0.001). Among the comorbidities present, the only statistically significant difference between the groups related to the presence of arterial hypertension. Arterial hypertension was significantly more prevalent in the overweight and obese patient group (*p* < 0.001). All patients who met the inclusion criteria underwent the RM according to the study protocol. No participants were excluded based on the exclusion criteria, and every patient underwent all the planned RMs without any complications, remaining stable throughout.

**Table 2:** Parameters of respiratory mechanics during all stages of the laparoscopic cholecystectomy and after the RM.

	Time	Normal-Weight Group I BMI 18.5–24.9 (*n* = 33)	Pre-Obese and Obese Class I Group II BMI 25.0–34.9 (*n* = 63)	*p*-Value
Ppeak (cmH_2_O)	T1	13.15 ± 2.24	15.63 ± 2.82	<0.001
	T2	17.09 ± 2.54	20.08 ± 2.87	<0.001
	T3	18.70 ± 2.52	21.08 ± 2.44	<0.001
	T4	20.00 ± 2.06	21.98 ± 2.42	<0.001
	T5	17.55 ± 1.84	20.19 ± 2.36	<0.001
	T6	15.97 ± 1.99	17.79 ± 1.89	<0.001
Pplat (cmH_2_O)	T1	10.15 ± 2.09	12.37 ± 2.55	<0.001
	T2	14.00 ± 2.32	16.70 ± 2.61	<0.001
	T3	15.58 ± 2.42	17.63 ± 2.51	<0.001
	T4	17.30 ± 2.21	18.54 ± 2.24	0.008
	T5	14.33 ± 1.87	16.02 ± 1.75	<0.001
	T6	13.52 ± 1.80	14.92 ± 1.80	<0.001
Cdin (mL/cmH_2_O)	T1	60.09 ± 11.78	51.04 ± 11.95	0.001
	T2	41.86 ± 6.84	36.70 ± 7.13	0.001
	T3	45.26 ± 8.14	39.03 ± 6.75	<0.001
	T4	46.50 ± 6.81	41.87 ± 7.49	0.004
	T5	60.89 ± 12.31	50.49 ± 10.27	<0.001
	T6	68.56 ± 12.42	59.27 ± 11.24	<0.001
Cstat (mL/cmH_2_O)	T1	72.58 ± 12.92	61.51 ± 13.95	<0.001
	T2	49.73 ± 8.95	43.50 ± 8.20	0.001
	T3	53.41 ± 11.10	46.12 ± 7.87	<0.001
	T4	55.06 ± 8.68	50.71 ± 9.60	0.032
	T5	73.34 ± 13.51	62.26 ± 12.41	<0.001
	T6	81.10 ± 13.34	70.18 ± 13.12	<0.001
DP (cmH_2_O)	T1	7.15 ± 2.09	9.37 ± 2.55	<0.001
	T2	11.00 ± 2.32	13.70 ± 2.61	<0.001
	T3	10.58 ± 2.42	12.63 ± 2.51	<0.001
	T4	10.30 ± 2.21	11.54 ± 2.24	0.008
	T5	7.33 ± 1.87	9.02 ± 1.75	<0.001
	T6	6.52 ± 1.80	7.92 ± 1.80	<0.001

T1–T6: T1—after induction of anesthesia and before the formation of pneumoperitoneum; T2—after the formation of pneumoperitoneum and positioning of the patient in the reverse Trendelenburg position; T3—after RM with PEEP values of 5 cm H_2_O; T4—after RM with PEEP values of 7 cm H_2_O; T5—after deflation and PEEP values of 7 cm H_2_O; T6—before waking with RM and PEEP values of 7 cm H_2_O; Ppeak—peak pressure; Pplat—plateau pressure; Cdin—dinamic compliance; Cstat—static compliance; DP—driving pressure.

**Table 3:** Changes in the Cstat value of non-obese and obese individuals at different time points: (*n* = 96; $\bar{x}$ ± SD).

Time Interval	Normal-Weight	Overweight and Obese
Δ2–Δ1	22.85 ± 8.09	18.00 ± 8.39 *
Δ3–Δ2	3.68 ± 5.35	2.62 ± 4.30
Δ4–Δ3	1.65 ± 7.15	4.59 ± 4.76
Δ5–Δ4	18.28 ± 7.40	11.55 ± 8.49 *
Δ6–Δ5	7.77 ± 7.78	7.92 ± 6.30

* *p* < 0.01.

**Table 4:** Arterial blood gas analysis: 1. before the recruitment maneuver and 2. after the second recruitment maneuver in group I (*n* = 33) and group II (*n* = 63) ($\bar{x}$ ± SD).

Variable	Normal-Weight (*n* = 33)	Overweight/Obese (*n* = 63)	*p*-Value
1. pH before RM	7.37 ± 0.04	7.39 ± 0.04	0.016
2. pH after RM	7.36 ± 0.04	7.37 ± 0.04	0.111
1. PaO_2_ before RM	200.79 ± 45.22	151.98 ± 41.84	<0.001
2. PaO_2_ after RM	212.82 ± 33.67	173.19 ± 39.00	<0.001
1. P/F ratio before RM	429.18 ± 100.14	330.52 ± 88.62	<0.001
2. P/F ratio after RM	457.88 ± 76.38	377.87 ± 83.86	<0.001
1. PaCO_2_ before RM	39.36 ± 3.19	38.83 ± 3.38	0.452
2. PaCO_2_ after RM	39.21 ± 3.60	39.48 ± 3.38	0.723
1. Lactate before RM	0.94 ± 0.25	0.96 ± 0.22	0.629
2. Lactate after RM	0.98 ± 0.26	1.00 ± 0.24	0.707
1. HCO_3_^−^ before RM	22.86 ± 2.21	23.94 ± 2.80	0.059
2. HCO_3_^−^ after RM	22.42 ± 2.39	26.25 ± 24.91	0.381
1. BE before RM	−2.08 ± 2.63	−1.07 ± 2.78	0.088
2. BE after RM	−3.06 ± 2.89	−2.09 ± 2.78	0.113
1. A-aDO_2_ before RM	84.62 ± 49.28	125.64 ± 39.71	<0.001
1. A-aDO_2_ after RM	71.94 ± 39.27	102.58 ± 38.33	0.001
1. paO_2_/pAO_2_ before RM	0.71 ± 0.17	0.55 ± 0.14	<0.001
2. paO_2_/pAO_2_ after RM	0.75 ± 0.13	0.63 ± 0.14	<0.001

**Table 5:** Arterial blood gas analysis in all the study participants combined for O_2_ pressure (PaO_2_): 1. before the recruitment maneuver and 2. after the recruitment maneuver in both groups (*n* = 96).

Variable	Mean	Standard Deviation	Standard Error
1. PaO_2_	168.76	48.73	4.97
2. PaO_2_	186.81 *	41.62	4.25

* *p* < 0.001.

**Table 6:** Heart rate value of subjects in both groups at different time points (*n* = 96).

Time	$\bar{x}$ ± SD	25th Percentile	50th Percentile	75th Percentile
T1. after induction of anest.	83.48 ± 14.62	72.25	81.50	93.75
T2. after pneumoperitoneum	77.46 ± 13.17	68.25	77.00	86.75
T3. after RM with PEEP5	74.57 ± 13.24	65.00	72.00	82.75
T4. after RM with PEEP7	74.15 ± 13.46	64.00	72.50	83.75
T5. after deflation	68.90 ± 12.09	62.00	67.50	75.00
T6. after RM at the end	67.50 ± 11.42	59.00	66.00	75.00

*p* < 0.001.

**Table 7:** Mean arterial blood pressure value of subjects in both groups at different time points (*n* = 96).

Time	$\bar{x}$	25th Percentile	50th Percentile	75th Percentile
T1. after induction of anest.	95.96 ± 14.80	85.00	95.00	107.75
T2. after pneumoperitoneum	100.89 ± 16.12	90.00	100.00	112.00
T3. after RM with PEEP5	105.03 ± 17.59	93.50	104.00	118.00
T4. after RM with PEEP7	103.76 ± 15.53	93.00	102.00	115.00
T5. after deflation	99.29 ± 14.12	90.00	97.00	109.75
T6. after RM at the end	94.70 ± 13.31	86.00	92.00	104.00

## Data Availability

The raw data supporting the conclusions of this article will be made available by the authors on request.

## References

[1] Young C.C., Harris E.M., Vacchiano C., Bodnar S., Bukowy B., Elliott R.R.D., Migliarese J., Ragains C., Trethewey B., Woodward A. (2019). Lung-protective ventilation for the surgical patient: International expert panel-based consensus recommendations. Br. J. Anaesth..

[2] Hess D.R. (2014). Respiratory mechanics in mechanically ventilated patients. Respir. Care.

[3] Banks J.A. (2018). Efficacy of Alveolar Recruitment Maneuvers in the Adult Obese Patient Undergoing General Anesthesia: A Systematic Review of the Literature. Master’s Thesis.

[4] Li X., Ni Z.-L., Wang J., Liu X.-C., Guan H.-L., Dai M.-S., Gao X., Zhou Y., Hu X.-Y., Sun X. (2022). Effects of individualized positive end-expiratory pressure combined with recruitment maneuver on intraoperative ventilation during abdominal surgery: A systematic review and network meta-analysis of randomized controlled trials. J. Anesth..

[5] Elokada S.A., Farag H.M. (2019). Preemptive Alveolar Recruitment Maneuver Followed by PEEP in Obese Patients Undergoing Laparoscopic Gastric Banding. Does it make a Difference? A Randomized Controlled Clinical Study. Open Anesth. J..

[6] Wynn-Hebden A., Bouch D.C. (2020). Anaesthesia for the obese patient. BJA Educ..

[7] Bluth T., Neto A.S., Schultz M.J., Pelosi P., de Abreu M.G. (2019). Effect of Intraoperative High Positive End-Expiratory Pressure (PEEP) with Recruitment Maneuvers vs Low PEEP on Postoperative Pulmonary Complications in Obese Patients: A Randomized Clinical Trial. JAMA-J. Am. Med. Assoc..

[8] Tomescu D.R., Popescu M., Dima S.O., Bacalbașa N., Bubenek-Turconi Ș. (2017). Obesity is associated with decreased lung compliance and hypercapnia during robotic assisted surgery. J. Clin. Monit. Comput..

[9] Kock K.S., Maurici R. (2018). Respiratory mechanics, ventilator-associated pneumonia and outcomes in intensive care unit. World J. Crit. Care Med..

[10] Chen L., Grieco D.L., Beloncle F., Chen G.-Q., Tiribelli N., Madotto F., Fredes S., Lu C., Antonelli M., Mercat A. (2022). Partition of respiratory mechanics in patients with acute respiratory distress syndrome and association with outcome: A multicentre clinical study. Intensive Care Med..

[11] Amato M.B.P., Meade M.O., Slutsky A.S., Brochard L., Costa E.L.V., Schoenfeld D.A., Stewart T.E., Briel M., Talmor D.S., Mercat A. (2015). Driving Pressure and Survival in the Acute Respiratory Distress Syndrome. N. Engl. J. Med..

[12] Brower R.G., Matthay M.A., Morris A., Schoenfeld D., Thompson B.T., Wheeler A., Acute Respiratory Distress Syndrome Network (2000). Ventilation with lower tidal volumes as compared with traditional tidal volumes for acute lung injury and the acute respiratory distress syndrome. N. Engl. J. Med..

[13] Lucangelo U., Bernabé F., Blanch L. (2005). Respiratory mechanics derived from signals in the ventilator circuit. Respir. Care.

[14] Meier A., Sell R.E., Malhotra A. (2020). Driving Pressure for Ventilation of Patients with Acute Respiratory Distress Syndrome. Anesthesiology.

[15] Reber A., Engberg G., Sporre B., Kviele L., Rothen H.U., Wegenius G., Nylund U., Hedenstierna G. (1996). Volumetric analysis of aeration in the lungs during general anaesthesia. Br. J. Anaesth..

[16] Whalen F.X., Gajic O., Thompson G.B., Kendrick M.L., Que F.L., Williams B.A., Joyner M.J., Hubmayr R.D., Warner D.O., Sprung J. (2006). The effects of the alveolar recruitment maneuver and positive end-expiratory pressure on arterial oxygenation during laparoscopic bariatric surgery. Anesth. Analg..

[17] Pelosi P., Croci M., Ravagnan I., Tredici S., Pedoto A., Lissoni A. (1998). The effects of body mass on lung volumes, respiratory mechanics, and gas exchange during general anesthesia. Anesth Analg..

[18] Steier J., Jolley C.J., Seymour J., Roughton M., Polkey M.I., Moxham J. (2009). Neural respiratory drive in obesity. Thorax.

[19] Pelosi P., Foti G., Cereda M., Vicardi P., Gattinoni L. (1996). Effects of carbon dioxide insufflation for laparoscopic cholecystectomy on the respiratory system. Anaesthesia.

[20] Sprung J., Whalley D.G., Falcone T., Warner D.O., Hubmayr R.D., Hammel J. (2002). The impact of morbid obesity, pneumoperitoneum, and posture on respiratory system mechanics and oxygenation during laparoscopy. Anesth. Analg..

[21] Talab H.F., Zabani I.A., Abdelrahman H.S., Bukhari W.L., Mamoun I., Ashour M.A., Bin Sadeq B., El Sayed S.I. (2009). Intraoperative ventilatory strategies for prevention of pulmonary atelectasis in obese patients undergoing laparoscopic bariatric surgery. Anesth. Analg..

[22] Andersson L.E., Bååth M., Thörne A., Aspelin P., Odeberg-Wernerman S. (2005). Effect of carbon dioxide pneumoperitoneum on development of atelectasis during anesthesia, examined by spiral computed tomography. Anesthesiology.

[23] Sharma K.C., Brandstetter R.D., Brensilver J.M., Jung L.D. (1996). Cardiopulmonary physiology and pathophysiology as a consequence of laparoscopic surgery. Chest.

[24] Futier E., Constantin J.-M., Pelosi P., Chanques G., Kwiatkoskwi F., Jaber S., Bazin J.-E. (2010). Intraoperative recruitment maneuver reverses detrimental pneumoperitoneum-induced respiratory effects in healthy weight and obese patients undergoing laparoscopy. Anesthesiology.

[25] Pei S., Wei W., Yang K., Yang Y., Pan Y., Wei J., Yao S., Xia H. (2022). Recruitment Maneuver to Reduce Postoperative Pulmonary Complications after Laparoscopic Abdominal Surgery: A Systematic Review and Meta-Analysis. J. Clin. Med..

[26] Almarakbi W.A., Fawzi H.M., Alhashemi J.A. (2009). Effects of four intraoperative ventilatory strategies on respiratory compliance and gas exchange during laparoscopic gastric banding in obese patients. Br. J. Anaesth..

[27] Hartland B.L., Newell T.J., Damico N. (2015). Alveolar recruitment maneuvers under general anesthesia: A systematic review of the literature. Respir. Care.

[28] Gattinoni L., Marini J.J., Pesenti A., Quintel M., Mancebo J., Brochard L. (2016). The “baby lung” became an adult. Intensive Care Med..

[29] Chen L., Del Sorbo L., Grieco D.L., Junhasavasdikul D., Rittayamai N., Soliman I., Sklar M.C., Rauseo M., Ferguson N.D., Fan E. (2020). Potential for Lung Recruitment Estimated by the Recruitment-to-Inflation Ratio in Acute Respiratory Distress Syndrome. A Clinical Trial. Am. J. Respir. Crit. Care Med..

[30] Gattinoni L., Carlesso E., Cressoni M. (2015). Selecting the ‘right’ positive end-expiratory pressure level. Curr. Opin. Crit. Care.

[31] Mazzinari G., Diaz-Cambronero O., Alonso-Iñigo J.M., Garcia-Gregorio N., Ayas-Montero B., Ibañez J.L., Neto A.S., Ball L., de Abreu M.G., Pelosi P. (2020). Intraabdominal Pressure Targeted Positive End-expiratory Pressure during Laparoscopic Surgery: An Open-label, Nonrandomized, Crossover, Clinical Trial. Anesthesiology.

[32] Güldner A., Kiss T., Serpa Neto A., Hemmes S.N., Canet J., Spieth P.M., Rocco P.R., Schultz M.J., Pelosi P., Gama de Abreu M. (2015). Intraoperative protective mechanical ventilation for prevention of postoperative pulmonary complications: A comprehensive review of the role of tidal volume, positive end-expiratory pressure, and lung recruitment maneuvers. Anesthesiology.

[33] Neto A.S., Hemmes S.N.T., Barbas C.S.V., Beiderlinden M., Fernandez-Bustamante A., Futier E., Gajic O., El-Tahan M.R., Al Ghamdi A.A., Günay E. (2016). PROVE Network Investigators. Association between driving pressure and development of postoperative pulmonary complications in patients undergoing mechanical ventilation for general anaesthesia: A meta-analysis of individual patient data. Lancet Respir. Med..

[34] del Portillo I.P., Vázquez S.T., Mendoza J.B., Moreno R.V. (2014). Oxygen Therapy in Critical Care: A Double Edged Sword. Health.

[35] Pelosi P., Ravagnan I., Giurati G., Panigada M., Bottino N., Tredici S., Eccher G., Gattinoni L. (1999). Positive end-expiratory pressure improves respiratory function in obese but not in normal subjects during anesthesia and paralysis. Anesthesiology.

[36] Myatra S.N. (2019). Hemodynamic effects of alveolar recruitment maneuvres in the operating room: Proceed with caution. J. Anaesthesiol. Clin. Pharmacol..

[37] Lovas A., Szakmány T. (2015). Haemodynamic Effects of Lung Recruitment Manoeuvres. Biomed. Res. Int..

[38] Biais M., Lanchon R., Sesay M., Le Gall L., Pereira B., Futier E., Nouette-Gaulain K. (2017). Changes in Stroke Volume Induced by Lung Recruitment Maneuver Predict Fluid Responsiveness in Mechanically Ventilated Patients in the Operating Room. Anesthesiology.

[39] Simon P., Girrbach F., Petroff D., Schliewe N., Hempel G., Lange M., Bluth T., de Abreu M.G., Beda A., Schultz M.J. (2021). Individualized versus Fixed Positive End-expiratory Pressure for Intraoperative Mechanical Ventilation in Obese Patients: A Secondary Analysis. Anesthesiology.

